# Knowledge, attitude, and practice of sexual healthcare and its influencing factors among oncology nurses: a multicenter study

**DOI:** 10.1093/sexmed/qfad001

**Published:** 2023-03-10

**Authors:** Jianfei Xie, Yi Zhou, Xiaofei Luo, Qinqin Cheng, Yating Luo, Yue Kang, Ziyu Wan, Panpan Xiao, Xing Zhou, Xiangyu Liu, Yinglong Duan, Andy SK Cheng

**Affiliations:** Nursing Department, The Third Xiangya Hospital, Central South University, Changsha, Hunan, China; Xiangya Nursing School, Central South University, Changsha, Hunan, China; Xiangya Nursing School, Central South University, Changsha, Hunan, China; Xiangya Nursing School, Central South University, Changsha, Hunan, China; Affiliated Cancer Hospital of School of Medicine Central South University Xiangya: Hunan Cancer Hospital, Changsha, Hunan, China; Xiangya Nursing School, Central South University, Changsha, Hunan, China; Xiangya Nursing School, Central South University, Changsha, Hunan, China; Xiangya Nursing School, Central South University, Changsha, Hunan, China; Xiangya Nursing School, Central South University, Changsha, Hunan, China; Xiangya Nursing School, Central South University, Changsha, Hunan, China; Affiliated Cancer Hospital of School of Medicine Central South University Xiangya: Hunan Cancer Hospital, Changsha, Hunan, China; Nursing Department, The Third Xiangya Hospital, Central South University, Changsha, Hunan, China; Department of Rehabilitation Sciences, Hong Kong Polytechnic University, Hong Kong, China

**Keywords:** oncology nursing, sexual health, knowledge, attitude, professional practice

## Abstract

**Background:**

The provision of sexual healthcare plays an integral role in the field of oncology nursing. However, limitations in the knowledge, attitude, and practice perspectives of oncology nurses require detailed study.

**Aim:**

In this study the authors sought to describe the knowledge, attitude, and practice of oncology nurses regarding sexual healthcare from a nationwide perspective and to explore the factors that influence them.

**Methods:**

This was a cross-sectional descriptive study using stratified random sampling of certified oncology nurses from 55 hospitals in 6 provinces in Central South China. In total, 2530 nurses participated and completed the Knowledge, Attitude and Practice questionnaire of Sexual Health Care (KAP of SHC), the Nurses Clinic Communication Competency Scale, the Nurses Professional Values Scale, and the General Self-efficacy Scale. Multivariate linear regression was used to explore influencing factors.

**Outcomes:**

The primary variable was the knowledge, attitude, and practice of sexual healthcare provision. Secondary variables included professional value, clinical communication competency, self-efficacy, and demographic factors.

**Results:**

The median KAP of SHC score was 139 (possible range 72 to 288). Attitude of SHC scored highest, followed by knowledge and practice scores. Professional values were positively associated with knowledge (odds ratio [OR] = 0.057; 95% CI: 0.023–0.091; *P* < .01) and attitude (OR = 0.319; 95% CI: 0.268–0.370, *P* < .01) of SHC. Clinical communication competency was only positively related to the attitude of SHC (OR = 3.960; 95% CI: 2.701–5.218, *P* < .01). Self-efficacy was positively related to KAP and the knowledge (OR = 0.616; 95% CI: 0.506–0.725, *P* < .01), attitude (OR = 0.187; 95% CI: 0.052–0.322, *P* < .01), and practice (OR = 0.840; 95% CI: 0.735-0.944, *P* < .01) of SHC.

**Clinical Implications:**

Knowledge assistance, attitude training, and practice coaching resources must be on the agenda to optimize professional practice for oncology nurses.

**Strengths and Limitations:**

This study provides data based on the Knowledge, Attitude, Belief and Practice (KABP) model in a nationwide sample of oncology nurses. In addition, the relationship between self-efficacy and KAP of SHC has been explored for the first time. The limitations are that this study may have some bias and did not take into account mediating relationships.

**Conclusions:**

Oncology nurses exhibit moderate levels of KAP of SHC. It is noteworthy that self-efficacy and position are the only 2 factors that influenced all aspects of KAP of SHC.

## Introduction

The provision of sexual healthcare (SHC) has been considered an integral component of patient care since the World Health Organization described sexual health.^1^ As stated by the Oncology Nursing Society, sexual health plays an essential role in the quality care and outcome standards of practical cancer care. It is clear that oncology-certified nurses are in a strategic position to provide SHC.^2^ In contrast to medical or surgical nursing staff, oncology nurses are open and positive about sexuality issues,^3^^,^^4^ which does not equate to an efficient response to patient sexual health concerns. Hesitancy to talk about sexuality, limited existing knowledge, and lack of experience in caring for patient sexuality issues are possible causes.^5–8^ In general, oncology nurses need to have deep knowledge and exercise reasonable judgment and a high degree of sensitivity when dealing with sexual health needs. This imbalance between high demands and lack of competence can lead to neglect in the area of sexuality in cancer nursing. Due to suboptimal knowledge, attitude, and practice (KAP) of SHC in oncology care, latent problems may persist and prolong, even affecting patient relationships and leading to noncompliance with treatment regimens, further compromising recovery and quality of life. Studies have supported the importance of providing sexual health services to patients,^9^^,^^10^ but the specifics and influencing factors of the KAP perspective have not been fully explored.

The professional values of nurses are defined as representing an ideal set of professional ethics whose interpretation and application are central to nursing practice.^11^ In a study by William et al,^12^ professional values were shown to be a significant predictor of nurses whi provide more psychosexual care to women diagnosed with gynecologic cancer. Clinical communication competency in nursing is defined as patient-centered exchange of information with patients, their families, and other health professionals to restore and promote the patient health and meet their needs.^13^ Effective communication contributes to the ability of nurses to empathize, especially based on sex-sensitive considerations.^14^ Providing patient-centered sexual communication has proven to be associated with the the attitudes and even the clinical practices of the health professional.^15^ Self-efficacy has been defined as a rather specific type of expectation that refers to an individual’ s beliefs and ability to perform specific behavior(s) to achieve a certain outcome.^16^ Self-efficacy has been identified to be positively associated with sexual health knowledge.^17^ To reduce sexual health disparities, it is the responsibility of nursing managers to ensure that nurses in clinical settings receive self-efficacy development regarding SHC education.^18^

Clearly, this study is conceptually based on the Knowledge, Attitude, Belief and Practice (KABP) model, the conceptual core of which is the cascade of knowledge, beliefs, and behaviors.^19^ Knowledge lays a solid foundation for behavior changes, while beliefs guide behavior change. When applied to clinical care, this process can be broken down into 3 consecutive components: knowledge, attitude, and practice, also known as KAP, which are then incorporated into the actual disease care process. Better knowledge can lead to positive attitudes, which in turn lead to good practice.^20^^,^^21^ It is currently known that a significant portion of oncology nursing research focuses only on knowledge and clinical practices in SHC.^22^ Approaches that consider attitudes as a whole may receive much less attention. Also, results regarding self-efficacy and confidence are inconsistent or not tested at all. This, coupled with unhelpful beliefs about SHC, exacerbates the lack of professional self-confidence. Whether professional values, clinical communication skills, and self-efficacy affect SHC attitudes or even overall KAP are also questions worth exploring. Furthermore, these issues have rarely been characterized in mainland China,^23-25^ and existing studies have been conducted in a limited scopes and with small sample sizes.^26^^,^^27^ Therefore, the main objectives of this study include the following: (1) to describe the current KAP for providing SHC, and (2) to explore the impact of professional values, clinical communication competency, self-efficacy, and demographic factors of the KAP of the oncology nurses for SHC provision. WE hypothesized that oncology nurses would have low KAP scores in addressing sexual wellness.

## Methods

### Study design and sampling

This study was a cross-sectional design that used the STROBE statement checklist. In this study, stratified random sampling was used based on hospital classification and regional distribution in the south-central region of China, which consists of 6 provinces, Henan, Hubei, Hunan, Guangdong, Guangxi, and Hainan. Stratified random sampling is to first divide the overall units into various types (or strata) according to certain criteria, and then to determine the number of sample units from each type according to the ratio of the number of units of each type to the number of units overall, and finally, to draw a sample from each type according to the random principle. Overall units are divided into various types (or strata) according to certain criteria, and then the number of sample units from each type is determined according to the ratio of the number of units of each type to the overall number of units. Finally, samples are drawn randomly from each type. First, the total number of oncology hospitals as of December 1, 2018, was extracted from all hospitals in Central South China; then, the number of tertiary, secondary, and other hospitals in each province was extracted. Second, the ratios of the total number of potentially tested oncology hospitals to the 3 levels of hospitals were calculated separately. Finally, hospitals of each level were randomly selected from each province using the respective ratios. Between January and July 2019, 55 hospitals (30 tertiary hospitals and 25 secondary and lower hospitals) were randomly selected as the sample based on the list of oncology hospitals. All data were obtained from the website of the National Health Commission of the People’s Republic of China (http://www.nhc.gov.cn/wjw/index.shtml). Tertiary hospitals were defined as specialized medical and preventive technology centers with higher education and research capabilities (at least 500 beds). Secondary hospitals are referred to as regional medical and preventive technology centers (100-499 beds).^28^

After the pilot hospitals were identified, the project leaders contacted the management of each hospital through the communication platform of the Chinese Nursing Association. Each administration promoted and invited oncology nurses who met the inclusion criteria and agreed to participate. Each recruited subject was sent a link to a questionnaire assessing sociodemographic information, knowledge, attitudes, and practices regarding the provision of sexual healthcare, communication skills, professional values, and self-efficacy. It took 15-30 minutes to complete the questionnaire. In the end, 2530 nurses participated and completed the questionnaire (response rate of 90.4%).

Inclusion criteria were as follows: (1) registered nurses working in a certified oncology department or other department that receives and treats more than 50% of cancer patients throughout the year, (2) more than 6 months of experience caring for cancer patients, and (c) speaking Chinese. Exclusion criteria were as follows: nurses who (1) worked uninterruptedly during the survey period, (2) experienced a major stressful event, or (3) had a serious physical or mental illness.

The study was initiated after approval by the Institutional Review Board (IRB) of Third Xiangya Hospital of Central South University, and written and verbal informed consent was obtained from each participant. The information and answers of each potential participant were evaluated under conditions of complete anonymity and strict confidentiality.

## Measurements

### Sociodemographic information

Information obtained for each participant included hospital type, hospital level, sex, age, years in the oncology department, position, education level, marital status, and number of children.

### The KAP of SHC

The Chinese version of the KAP questionnaire of SHC (KAP of SHC) was translated from the Dutch version^3^ and revised from the KABP model. Surveys based on the KABP model are commonly used to identify knowledge gaps and behavioral patterns in order to assess overall competence and take effective measures. The questionnaire consists of 24 items scored on a 4-point Likert scale. The 3 dimensions (knowledge, attitude, and practice) were used for each of the 24 entries but with different options (Table 1). In detail, subjects rated the adequacy of their knowledge and skills related to each entry from “inadequate” (1 point) to “more than adequate” (4 points), and the importance of each item from “not important” (1 point) to “very important” (4 points), and determined their practice of each item from “never” (1 point) to “almost always” (4 points). Each subscale had the same total score (range 24–96), and the total KAP score of SHC was the sum of the 3 subscales, ranging from 72 to 288. Higher scores indicated greater knowledge, positive attitudes, and better practices. The Chinese version was tested before this study and showed good validity (scale content validity index = 0.969) and high internal consistency (Cronbach α coefficient = 0.986).

**Table 1:** Score sheet for the KAP of SHC.

**Statement**	**KAP score**			
	**Inadequate/not important/never**			**More than adequate/very important/almost always**
1. Providing care for problems with sexual functioning	1	2	3	4
2. Asking questions about sexuality and sexual functioning	1	2	3	4
3. Determining care patient wants for sexuality and illness issues	1	2	3	4
4. Providing care for problems associated with changes in social contacts	1	2	3	4
5. Providing care for problems associated with changes in self-concept	1	2	3	4
6. Providing care for problems associated with changes in ability to find sex partner	1	2	3	4
7. Providing care for problems associated with emotional relationship patient/partner	1	2	3	4
8. Providing care for problems associated with changes in love-making (all activities except intercourse)	1	2	3	4
9. Providing care for problems associated with changes in intercourse	1	2	3	4
10. Providing care for problems associated with changes in masturbation	1	2	3	4
11. Planning care for sexual difficulties with nurses	1	2	3	4
12. Planning care for sexual difficulties with other professionals	1	2	3	4
13. Giving information/instruction about hygiene and physical care before/during/after sexual activities	1	2	3	4
14. Giving information/instruction about catheter and incontinence care before/during/after sexual activities	1	2	3	4
15. Giving information/instruction about stoma care before/during/after sexual activities	1	2	3	4
16. Giving information/instruction about illness-related reproductive issues	1	2	3	4
17. Giving information/instruction about managing illness-related symptoms or problems limiting sexual activities	1	2	3	4
18. Giving information/instruction over positions for intercourse given physiological and other limitations	1	2	3	4
19. Giving psychosocial support and counseling for self-concept concerns	1	2	3	4
20. Providing privacy for intimacy and sexuality within inpatient setting	1	2	3	4
21. Giving psychosocial support and counseling for difficulties associated with lovemaking, intercourse, masturbation	1	2	3	4
22. Giving psychosocial support and counseling for concerns about sexual attractiveness	1	2	3	4
23. Referring patients with sexual difficulties to other professionals	1	2	3	4
24. Evaluating and revising nursing care for sexual problems	1	2	3	4

KAP, Knowledge, Attitude, and Practice questionnaire.

### Communication competency

Communication competency was evaluated with the Nurses’ Clinic Communication Competency Scale (NCCCS).^13^ The scale has 58 items and was developed and tested by Zeng.^13^ Subjects responded to each item from “very poor” (1 point) to “very good” (5 points). The total score ranged from 58 to 290, with higher scores indicating better communication levels. This scale consists of 6 areas: team communication competency (6 items), basic language communication competency (11 items), basic nonlanguage communication competency (7 items), emotional perception competency (9 items), emotional support competency (6 items) and communication competency in difficult clinical scenes (19 items). The mean value of the scale was used in this study, which ranged from 1 to 5. The scale had an overall Cronbach α of 0.978 and a dimensional range of 0.868–0.954. The scale had a coefficient of 0.923and a range of 0.765–0.916 for each dimension.

### Professional value

Professional value was evaluated using the Nurses Professional Values Scale (NPVS).^29^ The scale, originally developed by Gong et al, consists of 26 items addressing the following four dimensions: providing care (10 items), professional characteristics (7 items), reliance (6 items), and behaviorism (3 items). Each item was rated from “unimportant” (1 point) to “very important” (5 points). Total scores ranged from 26 to 130. Higher NPVS scores indicate greater professional value. The reliability of the retest was reported as 0.639. The overall Cronbach α was calculated as 0.959, with a range of 0.729 to 0.929 for each subscale.

### Self-efficacy

Self-efficacy was assessed with the Chinese version of the General Self-Efficacy Scale (GSES).^30^ The GSES has 10 items to measure self-efficacy. Each item is rated from 1 to 4 points (4 levels). A total score is calculated to indicate self-efficacy, with a high score indicating a higher level of self-efficacy. The scale has a Cronbach α coefficient of 0.87, with a retest reliability and split-half reliability of 0.83 and 0.82, respectively.

### Data analysis

Data were analyzed using the IBM Statistical Package for the Social Sciences version 26.0 (IBM Corp, Armonk, New York). Missing data were processed prior to analysis. If a variable was missing for a sample, the mean of this variable for all samples was filled in. Statistical analysis was performed in 2 steps in sequence: descriptive analysis and inferential analysis. Descriptive statistics included frequencies, percentages, medians, and percentile ranges (25th and 75th percentiles). The inferential process included the Mann-Whitney test, Kruskal-Wallis test, Spearman correlation, and multiple linear stepwise regression. To examine the factors significantly associated with the KAP of SHC, multiple linear stepwise regression was performed with the KAP score of SHC as the dependent variable and the variables with *P* < .05 in univariate analysis as independent variables.

## Results

### Demographic characteristics

The majority of the nurses were from general hospitals (89.3%) and tertiary hospitals (69.4%). Females constituted the majority (98.5%). In terms of age, more than 85% were under 40 years of age (86.2%). In terms of years of service, nurses were not in the minority in the first 5 years (54.0%) or the second 5 years (31.1%). Most of the subjects held the position of primary nurse (39.3%) or senior nurse (39.6%). Nurses with a Bachelor’s degree (73.3%) were in the majority. The majority of oncology nurses were married (72.3%), with 1 or 2 children, 28.6% and 33.6%, respectively (Table 2).

**Table 2:** Demographic variables and univariate analysis of the KAP of SHC (*n* = 2530).

**Demographic variables**	***n***	**%**	**KAP of SHC**	**Knowledge**	**Attitude**	**Practice**
			***Z*** ^**a**^ ***/H*** ^**b**^ ***(P)***	***Z*** ^**a**^ ***/H*** ^**b**^ ***(P)***	***Z*** ^**a**^ ***/H*** ^**b**^ ***(P)***	***Z*** ^**a**^ ***/H*** ^**b**^ ***(P)***
Hospital type						
Specialist	271	10.7	−2.604	−3.817	−.098	−3.467
Comprehensive	2259	89.3	.009^**^	<.001^***^	.922	<.001^***^
Hospital level						
Tertiary	1756	69.4	.856	4.407	3.507	5.011
Secondary	691	27.3	.652	.110	.173	.082
Others	83	3.3				
Sex						
Male	39	1.5	−2.377	−2.498	−1.833	−1.726
Female	2491	98.5	.017^*^	.012^*^	.067	.084
Age, years						
≤25	423	16.7	25.960	14.488	16.745	31.699
26–30	675	26.7	<.001^***^	.006^**^	.002^**^	<.001^***^
31–35	749	29.6				
36–40	334	13.2				
>40	349	13.8				
Years in oncology department						
<1	558	22.1	8.662	14.509	2.838	11.774
1–5	808	31.9	.070	.006^**^	.585	.019^**^
6–10	786	31.1				
11–15	258	10.2				
>15	120	4.7				
Position						
Junior nurse	352	13.9	51.026	33.604	24.132	41.326
Primary nurse	995	39.3	<.001^***^	<.001^***^	<.001^***^	<.001^***^
Senior nurse	1001	39.6				
Vice professor	182	7.2				
Education level						
Technical school	42	1.6	9.486	18.127	23.016	8.238
Junior college	541	21.4	.023^*^	<.001^***^	<.001^***^	.041^*^
Bachelor	1854	73.3				
Master and above	93	3.7				
Marital status						
Married	1828	72.3	13.741	7.490	11.199	3.188
Unmarried	654	25.8	<.001^***^	.024^*^	.004^*^	.203
Widowed/divorced	48	1.9				
Children, *n*						
None	956	37.8	2.061	1.336	1.683	1.882
1	725	28.6	.357	.513	.431	.390
2	849	33.6				

KAP of SHC, Knowledge, Attitude, and Practice questionnaire of Sexual Health Care.

^***^ *P <* .001.

^**^ *P* < .01.

^*^ *P* < .05.

a Mann-Whitney test.

b Kruskal-Wallis test.

### Scores of KAP of SHC, NCCCS, NPVS, and GSES

The median KAP of Chinese oncology nurses for SHC was 139.00 (Q_25_ = 116.00, Q_75_ = 167.00; Q_25_ indicates the twenty-fifth percentile, Q_75_ indicates the seventy-fifth percentile), representing a moderate level of knowledge, attitude, and practice of SHC. The dimension with the highest median score was attitude of SHC (median score = 66.00, Q_25_ = 48.00, Q_75_ = 72.00), and the lowest was practice of SHC (median score = 35.00, Q_25_ = 24.00, Q_75_ = 48.00). The median NCCCS score was 4.14 (Q_25_ = 3.93, Q_75_ = 4.95), and NPVS score was 104.00 (Q_25_ = 85.00, Q_75_ = 116.00), and GSES score was 30.00 (Q_25_ = 24.00, Q_75_ = 31.00) (Table 3).

**Table 3:** Scores of KAP of SHC, NCCCS, NPVS, and GSES (*n* = 2530).

**Dimension**	**Q**_**50**_ **(Q**_**25**_ **, Q**_**75**_ **)**	**Actual range**	**Possible range**	**Centesimal score**
KAP of SHC	139.00 (116.00, 167.00)	72.00–288.00	72.00–288.00	48.26
Knowledge	38.00 (27.00, 48.00)	24.00–96.00	24.00–96.00	39.58
Attitude	66.00 (48.00, 72.00)	24.00–96.00	24.00–96.00	68.75
Practice	35.00 (24.00, 48.00)	24.00–96.00	24.00–96.00	36.46
NCCCS	4.14 (3.93, 4.95)	1.00–5.00	1.00–5.00	82.80
NPVS	104.00 (85.00, 116.00)	26.00–130.00	26.00–130.00	80.00
GSES	30.00 (24.00, 31.00)	10.00–40.00	10.00–40.00	75.00

GSES, General Self-efficacy Scale; KAP of SHC, The Knowledge, Attitude and Practice questionnaire of Sexual Health Care; NCCCS, Nurses’ Clinic Communication Competency Scale; NPVS, Nurses Professional Values Scale; GSES, General Self-Efficacy Scale. Q_25_, twenty-fifth percentile, Q_50_, fiftieth percentile, Q_75_, seventy-fifth percentile.

### Univariate analysis

Nonparametric tests showed that hospital type, sex, age, years in the oncology department, position, education level, and marital status were associated with KAP of SHC (*P* < .05 for all; Table 2). According to the Spearman correlation coefficients (Table 4), knowledge of SHC was positively associated with NCCCS (*r*_s_ = 0.150, *P <* .001), NPVS (*r*_s_ = 0.188, *P* < .001), and GSES (*r*_s_ = 0.234, *P <* .001). Attitude of SHC was positively associated with NCCCS (*r*_s_ = 0.394, *P <* .001), NPVS (*r*_s_ = 0.444, *P <* .001), and GSES (*r*_s_ = 0.273, *P <* .001). Practice of SHC was positively associated with NCCCS (*r*_s_ = .056, *P <* .01), NPVS (*r*_s_ = 0.087, *P <* .001), and GSES (*r*_s_ = 0.203, *P* < .001). The KAP of SHC was positively correlated with NCCCS (*r*_s_ = 0.265, *P* < .001), the NPVS (*r*_s_ = 0.315, *P <* .001), and the GSES (*r*_s_ = 0.299, *P* < .001).

**Table 4:** Correlation between KAP of SHC, NCCCS, NPVS, and GSES.

	**KAP of SHC**	**Knowledge**	**Attitude**	**Practice**
**NCCCS**	.265^***^	.150^***^	.394^***^	.056^**^
**NPVS**	.315^***^	.188^***^	.444^***^	.087^***^
**GSES**	.299^***^	.234^***^	.273^***^	.203^***^

GSES, General Self-efficacy Scale; KAP of SHC, Knowledge, Attitude, and Practice questionnaire of Sexual Health Care; NCCCS, Nurses’ Clinic Communication Competency Scale; NPVS, Nurses Professional Values Scale.

^***^ *P* < .001.

^**^ *P* < .01

^*^ *P* < .05.

### Multiple linear stepwise regression of KAP of SHC and influencing factors

After adding the statistically significant variables from univariate analysis to multiple linear stepwise regression. This analysis revealed that GSES, position, hospital type, NPVS, marital status, and sex were significant factors associated with the knowledge aspect of SHC (F = 49.173, *P* < .001, adjusted *R*^2^ = 0.103). NPVS, NCCCS, position, GSES, and education level were significantly associated with the attitude aspect of SHC (F = 148.985, *P* < .001, adjusted *R*^2^ = 0.226). GSES, position, and hospital type were significantly associated with the practice aspect of SHC (F = 97.163, *P* < .001, adjusted *R*^2^ = 0.102). GSES, NPVS, position, hospital type, NCCCS, marital status, and sex were the main factors of the KAP of SHC (F = 82.232, *P* < .001, adjusted *R*^2^ = 0.186) (Table 5).

**Table 5:** Multiple stepwise regression for variables associated with the KAP of SHC.

**Model**	**B (95% CI)**	**SE**	**β**	**t**	***P***	**VIF**	**Adjusted *R*** ^**2**^
KAP of SHC							0.184
Constant	103.968 (74.266–133.671)	15.147		6.864	<.001^***^		
GSES	1.610 (1.312–1.908)	0.152	.229	10.600	<.001^***^	1.446	
NPVS	0.388 (0.276–0.500)	0.057	.178	6.793	<.001^***^	2.125	
Position	−7.594 (−9.658 to −5.530)	1.053	−.139	−7.215	<.001^***^	1.147	
Hospital type	−9.100 (−14.183 to −4.017)	2.592	−.063	−3.510	<.001^***^	1.005	
NCCCS	4.189 (1.415–6.962)	1.414	.074	2.961	.003^**^	1.949	
Marital status	−3.448 (−6.847 to −0.050)	1.733	−.038	−1.990	.047^*^	1.157	
Sex	−12.045 (−24.841 to 0.752)	6.526	−.033	−1.846	.065	1.010	
Knowledge of SHC							0.103
Constant	42.489 (31.512–53.466)	5.598		7.590	<.001^***^		
GSES	0.616 (0.506–0.725)	0.056	.247	11.050	<.001^***^	1.405	
Position	−2.165 (−2.932 to −1.397)	0.391	−.111	−5.531	<.001^***^	1.145	
Hospital type	−4.007 (−5.899 to −2.116)	0.965	−.078	−4.154	<.001^***^	1.005	
NPVS	0.057 (.023–0.091)	0.017	.074	3.305	<.001^***^	1.414	
Marital status	−1.778 (−3.042 to −0.513)	0.645	−.056	−2.757	.006^**^	1.157	
Sex	−5.020 (−9.782 to −0.258)	2.429	−.039	−2.067	.039^*^	1.010	
Attitude of SHC							0.226
Constant	9.106 (3.706–14.506)	2.754		3.307	<.001^***^		
NPVS	0.319 (0.268–0.370)	0.026	.314	12.266	<.001^***^	2.143	
NCCCS	3.960 (2.701–5.218)	0.642	.151	6.169	<.001^***^	1.948	
Position	−2.669 (−3.579 to −1.759)	0.464	−.105	−5.750	<.001^***^	1.082	
GSES	0.187 (0.052–0.322)	0.069	.057	2.720	.007^**^	1.440	
Education level	1.714 (0.279–3.149)	0.732	.043	2.343	.019^*^	1.108	
Practice of SHC							0.102
Constant	30.456 (25.020–35.893)	2.773		10.985	<.001^***^		
GSES	0.840 (0.735–0.944)	0.053	.297	15.704	<.001^***^	1.006	
Position	−2.771 (−3.587 to −1.956)	0.416	−.126	−6.661	<.001^***^	1.006	
Hospital type	−3.911 (−6.053 to −1.770)	1.092	−.068	−3.581	<.001^***^	1.001	

GSES, General Self-efficacy Scale; KAP of SHC, Knowledge, Attitude, and Practice questionnaire of Sexual Health Care; NCCCS, Nurses’ Clinic Communication Competency Scale; NPVS, Nurses Professional Values Scale; VIF, variance inflation factor.

^***^ *P* < .001

^**^ *P* < .01

^*^ *P* < .05.

KAP of SHC, *F* = 82.232, *P* < .001; knowledge of SHC, *F* = 49.173, *P* < .001; attitude of SHC, *F* = 148.985, *P* < .001; practice of SHC, *F* = 97.163, *P* < .001.

## Discussion

The level of KAP for SHC among Chinese oncology nurses appears to be moderate compared to the known information from the Dutch scale.^3^ In China, few studies have discussed the current status and factors associated with the KAP of SHC, and each instrument varies considerably in terms of implementation domain and clinical applicability (eg, self-designed questionnaire,^25^ Sexuality Attitudes and Beliefs Survey,^31^ and Sex Knowledge and Attitude Test^32^). It is difficult to compare the KAP levels of Chinese oncology nurses on SHC. However, it is still noteworthy that the scores for attitude were the highest among all dimensions (knowledge 38.00, attitude 66.00, practice 35.00). One possible reason for this finding is that despite the prevalence of barriers to discussing sexuality reported by oncology providers, they all perceive it as part of their responsibility.^33^ In fact, nurses on several oncology wards have realized that addressing sexuality issues is an important responsibility.^24^^,^^25^ Given the critical position of attitude in the KABP model, it is reasonable to view this trend in oncology as a symbol of potential but sustained improvement in the KAP of SHC scores of nurses. Given that deficits in professional judgment, inadequate communication skills, and lack of self-awareness are difficulties that prevent oncology nurses from adhering to SHC values,^12^^,^^22^^,^^34^ we suggest that factor analysis is a better approach.

Professional values were positively associated with knowledge and attitude toward the provision of SHC. Previous findings have shown that providing psychosexual services is influenced by professional values.^12^ According to the substantive theory, professional and personal values are considered to be dichotomous. The influence of personal values is a barrier to healthcare promotion, whereas being influenced by professional values and moral obligations helps to promote healthcare.^35^ These findings imply that an oncology nurse reconciles his/her personal beliefs derived from personal value systems or cultural backgrounds, and believes in the importance of holistic care when providing sexual health services, although nonjudgmentalism remains to be recognized and promoted in better practice.

The study results showed that clinical communication competency was positively associated only with the attitude of SHC provision. For oncology nurses, feelings of discomfort are representative attitudes toward SHC and are often associated with levels of communication capability.^36^ One potential explanation is that miscommunication may make staff fearful of putting the nurse-client relationship at risk. Furthermore, better communication skills lead to greater confidence, as low levels of professional confidence are often perceived as a barrier related to communication competency.^27^^,^^37^^,^^38^ Communication skills training has been identified as a core element of patient- and professional-oriented interventions.^39–41^ Therefore, it is necessary to establish a standard of communication that emphasizes the skillful role of the oncology nurses in communication.^39^

Our findings showed that self-efficacy was positively correlated to KAP and the knowledge, attitude, and practice dimensions of SHC. Notably, according to our narrative above, professional values and clinical communication also influenced the attitude dimension. In addition, attitude pertaining to SHC provision has been proven to explain the gap between the professional role and practice in identifying attitudinal barriers for oncology nurses.^42^ In consideration of the existing KABP model, information and knowledge are the basis for establishing positive attitudes, and attitudes are the driving force for behavior modification. It is reasonable that self-efficacy is not only related to attitude, but is also associated with knowledge and practice. Moreover, self-efficacy has previously been proven to be associated with perceived competency and confidence in coping with sexual health issues and may play an important role in communication skills.^43^ Also, in this previous study, perceived professional confidence and communication competency were factors that influenced sexual health service provision. It is therefore not surprising that self-efficacy is a main factor influencing KAP of SHC.

We found that oncology nurses in higher positions reported lower KAP scores of SHC, which is inconsistent with the results of a Korean study.^42^ A potential reason for this finding is that nurses in higher positions (eg, senior nurses and directors, 46.8% in this study) typically report much less direct client contact than staff nurses.^44^ Additionally, SHC scores for KAP among oncology nurses from different types of hospitals were significantly different. Notably, nurses in comprehensive hospitals scored lower on the KAP for providing SHC in specialist hospitals, unlike a previous study.^27^ One possible explanation is that comprehensive reform programs focus on management systems and regulatory mechanisms, which may increase the likelihood of overlooking issues that are not yet on the priority list.^45^^,^^46^ Moreover, we found that married oncology nurses tended to report higher scores on the KAP of SHC, which is consistent with a previous study.^47^ This finding added to the evidence that marital status is one of the potential influential factors in providing sexual healthcare in the oncology setting.

This study provides a set of suggestions for improving SHC provision. First, oncology nurses in China reported moderate levels of the KAP of SHC. Nursing administration needs to be aware of this issue, and knowledge assistance, attitude training, and practice coaching resources for healthcare professionals must be on the agenda. Second, professional value, clinical communication, and self-efficacy are positively associated with KAP of SHC, a finding that opens up new possibilities for oncology nurses, such as supporting beneficial professional value, asking about sexual health with clinical communication, and developing self-efficacy training programs. Third, nurses in higher positions and from comprehensive hospitals reported lower levels of KAP of SHC. To improve the accessibility and quality of sexual health education, measures specifically designed for oncology nurses in higher positions or comprehensive hospital are needed. Finally, staff education, while critical to the overall service approach, is deficient in implementing the intended sexual health agenda without the reinforcement of public health services. Therefore, ensuring clearer clinical psychosexual referral pathways from oncology health services is also critical.^48^

However, the study still has some limitations. First, although the oncology hospitals were randomly sampled, the participation of oncology nurses depended on voluntariness. Non-probability sampling may be one of the weaknesses of this study. Second, 98.5% of the participants in this study were female, which may result in some bias. Third, the study was based on self-reported data, which are inclined to be subjective, particularly for items on sensitive issues. Fourth, it remains unclear whether self-efficacy has an indirect effect on KAP of SHC mediated by professional value and/or clinical communication, and its possible mechanisms remain to be explored. Fifth, due to the cross-sectional descriptive design, inference of causal effects in this study is limited. However, to our knowledge, this survey provides, for the first time, substantial KAP data on SHC in a nationwide sample of Chinese oncology nurses. Second, this study provides a unique perspective based on the KABP model and support the validity of the model. Third, this is to our knowledge the first study to explore oncology nurses’ self-efficacy and KAP of SHC, adding important evidence to the relationship.

## Conclusion

In our study, oncology nurses reported a moderate level of KAP of SHC. Self-efficacy, professional values, position, hospital type, clinical communication, marital status, and sex were found to influence KAP of SHC provision. Self-efficacy and position were the only 2 factors that influenced all aspects of KAP of SHC for special needs medical services, which should be noted. The potential benefits of self-efficacy, professional values and clinical communication should be emphasized to effectively promote knowledge assistance, attitude training and practice coaching resources for oncology nurses.
